# Resistance Fighters: Developing a Novel HMG-CoA Reductase Inhibitor to Combat Gram-Positive Bacteria

**DOI:** 10.1063/4.0000852

**Published:** 2025-10-27

**Authors:** Phillip S Rushton, Calvin N Steussy, Sucharita Bose, Daneli Lopez-Perez, Tim Schmidt bachelors, Mohamed N Seleem, Mark Lipton, Cynthia V Stauffacher

**Affiliations:** 1 Purdue University, West Lafayette, IN, USA; 2 Institute for Stem Cell Science and Regenerative Medicine (DBT-inStem), Bengalore, Karnataka, India; 3 Food and Drug Administration, Silver Springs, MD, USA; 4 Virginia Tech, Blacksburg, VA, USA

## Abstract

Multi-drug resistant bacteria infect millions of people per year leading to billions of dollars in clinical costs and lowered quality of life. With fewer antibiotic drugs in development and fewer reserve antibiotics available to treat infections there is a growing need for drugs that target novel modes of action. With the discovery that the bacterial mevalonate pathway is essential for pathogenic gram-positive bacteria and possesses distinct elements from evolutionarily higher mevalonate pathways it was hypothesized that drugs could be designed to specifically target this pathway with a novel mode of action. This includes the CDC’s serious threat level ranked gram-positive strains of Vancomycin Resistant *Enterococcus faecalis* (VRE), Methicillin Resistant *Staphylococcus aureus* (MRSA), drug-resistant *Streptococcus pneumoniae* and Clostridioides difficile infections (CDI). Our lab has screened thousands of compounds against HMG-CoA reductase (HMGR), the rate limiting enzyme of the mevalonate pathway, which led to the discovery of a compound possessing low micromolar inhibitory constants against *E. faecalis* HMGR. Through iterative design phases guided by enzyme kinetics and X-ray crystal structures of HMGR bound with compounds it was possible to synthesize successive generations of better inhibitors. *In vivo* anti-bacterial experiments revealed many of these compounds can inhibit bacterial growth at low micromolar concentrations against VRA and MRSA strains. Recent work aims to expand the design parameters of the lead compound and/or generate new lead compounds by discovering smaller inhibitory chemical fragments that can be synthetically combined. A library of chemical fragments was screened against *E. faecalis* and *S. aureus* HMGR for enzyme inhibition. The best compounds were soaked into the more robust *Pseudomonas mevalonii* HMGR homolog crystals to evaluate possible modes of inhibition. Informed by these structures, a more effective antibiotic can be generated with lower inhibitory constants and effective bacterial control. This drug could be used to fight some of the worst bacterial infections humanity suffers.

